# Cover Picture: Nonlinear d^10^-ML_2_ Transition-Metal Complexes (ChemistryOpen 3/2013)

**DOI:** 10.1002/open.201390010

**Published:** 2013-06-20

**Keywords:** bond theory, density functional calculations, energy decomposition analysis, molecular geometry, transition-metal complexes, π backdonation

## Abstract

**Cover Picture:**

Lando P. Wolters and F. Matthias Bickelhaupt*

**The cover picture illustrates** the authors' quantum chemical finding that π electrons can significantly bend otherwise linear d^10^-ML_2_ complexes through backbonding. The foreground features a typical series of linear and nonlinear computed equilibrium geometries, while, in the background, one discerns quantitative numerical output of the bonding and energy decomposition analyses (EDA). The interpretation of the numerical data in terms of Kohn–Sham molecular orbital (MO) theory constitutes a predictive bonding model that explains the effects. The essence of this model has been sketched on paper during a brainstorming session (see the blue, hand-drawn orbital diagrams). It reveals that the second π-accepting ligand is, in a sense, hunting for “fresh” (not yet stabilized) d_π_ electrons. The insights obtained in this work are relevant not only for structural coordination chemistry but are envisaged to lead to applications in rational catalyst design. For more details, see the Full Paper by F. Matthias Bickelhaupt et al., on p. 106 ff.

